# Implications for Conservation of Collection of Mediterranean Spur-Thighed Tortoise as Pets in Morocco: Residents’ Perceptions, Habits, and Knowledge

**DOI:** 10.3390/ani10020265

**Published:** 2020-02-07

**Authors:** Amalia Segura, Miguel Delibes-Mateos, Pelayo Acevedo

**Affiliations:** 1 Instituto de Investigación de Recursos Cinegéticos, IREC (UCLM-CSIC-JCCM), Ronda de Toledo, 12, 13071 Ciudad Real, Spain; pelayo.acevedo@gmail.com; 2 Instituto de Estudios Sociales Avanzados (IESA-CSIC), Campo Santo de los Mártires 7, 14004 Córdoba, Spain; mdelibes@iesa.csic.es

**Keywords:** pet trade, questionnaire survey, social perception, *Testudo graeca*, Likert scale

## Abstract

**Simple Summary:**

The trading and collection of wildlife for pets is one of the main threats for the conservation of some species worldwide. Assessing the human dimension of it is essential to improve our understanding of its drivers, which may help inform the design of effective species conservation strategies. We address this issue using the Mediterranean spur-thighed tortoise (*Testudo graeca*) as a case study. This species has sharply declined in its native range, tortoise trade and non-commercial collection for pets being some of the main threats. In fact, both uses have been documented in southern Europe and northern Africa, although this species has been protected by the Convention on International Trade in Endangered species of Wild Fauna and Flora (CITES) since 1975. Our study, which was based on a questionnaire survey, (i) demonstrated that many people in Rabat city (Morocco) and surroundings keep tortoises as pets (55%; n = 480), most of which had been collected directly from wild populations, and (ii) highlighted the limited ecological and biological knowledge of tortoise owners (mainly of those living in the city of Rabat) on the species. Our findings evidence how both the sociological context and the role of consumers/harvesters play a major part in this problem with international projection. We discussed deeply how tortoise non-commercial collection might affect its conservation and welfare, and recommended appropriate actions that focus on strengthening collection bans.

**Abstract:**

The trading and collection of wild animals as pets may be cause for concern regarding animal welfare and species conservation. These concerns can be exemplified by Mediterranean spur-thighed tortoise (*Testudo graeca*), a long-living species whose use as pets is long established. The human dimension plays a major role in the wildlife for the pet collection, and is particularly important in countries like Morocco, where this might pose a threat to the conservation of the species involved. This study, which is based on a questionnaire survey (n = 480 participants), documents the fact that many people in Morocco keep tortoises as pets: 55% of the participants in the survey and over two tortoises/person. Importantly, most captive tortoises, particularly juveniles, had been collected directly from wild populations by their owners (42%, n = 264). In general, the tortoise owners had limited knowledge of their tortoises’ habits and requirements, although rural people were more likely to acknowledge that the tortoise is a wild and threatened species. Our study reveals that non-commercial collection is a common activity in Morocco that may threaten wild tortoise populations and hence species conservation, and it could have consequences regarding the welfare of the animals. We were also able to identify the profile of people towards whom education campaigns should be directed in order to reduce the number of tortoises collected from wild populations. Additional field research should also be conducted to quantify the impact of pet collection on wild tortoise populations.

## 1. Introduction

Most tortoises and turtles are struggling to persist in the modern world, a fact that is generally overlooked [1]. The main threats that these species are experiencing worldwide are habitat loss, climate change (because the sex of many tortoises and turtles is determined by their environment) and their unsustainable overexploitation as pets and food [2,3]. These factors are leading to steep declines in the populations of many tortoises and turtles, which are, therefore, confronted with a high risk of extinction and are consequently legally protected species (e.g., [4,5,6]).

The trading and collection of wildlife are cause for concern regarding animal welfare, public and health safety, species conservation, and ultimately environmental degradation [7,8]. In this respect, intentional harvest has been reported as the second largest threat to the survival of many reptiles [9], and the trading and collection of tortoises and turtles are considered the highest when compared to other reptiles [10,11]. Certain species and groups of tortoises and turtles, such as Testudines (which comprises 341 species) are preferred by pet owners over others [12], signifying that the likelihood that they are overexploited is higher. One of these species is the Mediterranean spur-thighed tortoise (*Testudo graeca*), which has traditionally been transported from North Africa to Europe to be sold as pets [13]. For example, in the 1960s 100,000 Mediterranean spur-thighed tortoises were introduced from Morocco to Europe per year [14], and Nijman and Bergin [15] recently observed large volumes of Mediterranean spur-thighed tortoises captured for illegal export in Morocco; the largest were found in the cities of Tangier and Rabat. Additionally, Brianti et al. [16] documented the illegal importation of 1400 tortoises from North Africa to Sicily in 2008.

Although the Mediterranean spur-thighed tortoise is primarily found in dense forests, grasslands and some coastal scrublands [17,18,19], its high adaptability to different environments means that this species is frequently sold as a resistant pet. Although tortoises are often found in markets, people also collect some animals from wild populations for personal use [20,21,22,23]. In addition, houses with gardens that are rented in cities and villages of Morocco often hold tortoises as pets (hereafter denominated as house heritage). The Mediterranean spur-thighed tortoise has been protected by the Convention on International Trade in Endangered species of Wild Fauna and Flora (CITES, Appendix 2) since 1975 and is also protected by Moroccan conservation laws like the King’s decree n° 1-11-84 promulgating Law n° 29-05 on the Protection of Species of Flora and Fauna and the Control of their Trade that bans its sale. In spite of this legal protection, the truth is that Mediterranean spur-thighed tortoises are currently sold openly in Morocco [23], collected illegally by owners [21], and are often exported to Europe, as mentioned above. In fact, human use seems to be one of the main factors jeopardizing the conservation of this tortoise species, which is Red-listed as Vulnerable by the International Union for Conservation of Nature [24].

Investigating the human dimension of the trading and collection of wildlife for pets is essential if we are to improve our understanding of its drivers, which will help inform the design of effective species conservation and management strategies [25]. In particular, it is necessary to attain more in-depth knowledge of pet owners’ values, perceptions, and attitudes towards wildlife pets, in addition to investigating their behavior in relation to their pets [26,27,28]. As an example, some survey studies in China and Vietnam have shown that people’s preferences for wild species as food or for traditional medicine are important drivers of the wildlife trade, which probably influence the market [26,27]. This need to understand the human dimension in the long-established use of wildlife as pets is especially important in countries like Morocco, not only because it is part of the Mediterranean basin hotspot, but also because the trading and collection of wildlife may simultaneously represent an important source of income for local people and be a threat to the conservation of the wildlife species involved [23].

In this study, we employed a questionnaire survey to assess (1) whether tortoise ownership is frequent in the city of Rabat and a smaller neighboring village, (2) tortoise owners’ perceptions, preferences and habits, and (3) their knowledge of the biological and ecological requirements of the species and its conservation status. This may help improve our understanding of the role played by non-commercial tortoise collection and its magnitude, and consequently its potential effects on wild tortoise populations. Given that perceptions and attitudes usually vary between urban and rural people [29], and that this has been particularly observed in the case of users of other tortoise species and areas [30], we also aimed to assess potential differences in the perceptions and knowledge of the Mediterranean spur-thighed tortoise between residents in the main city of Rabat and those living in a smaller neighboring rural village. Finally, we discuss the information gathered in terms of opportunities for tortoise conservation and welfare.

## 2. Materials and Methods

### 2.1. Study Area

**Figure 1:**
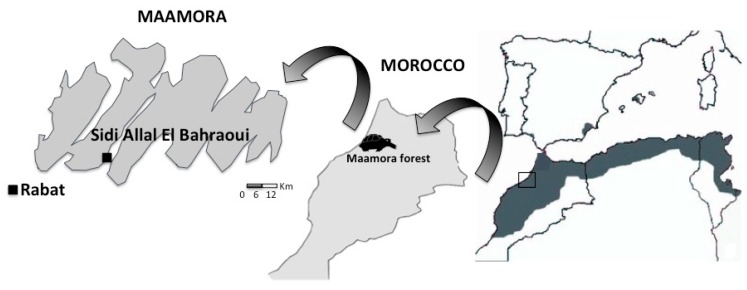
Distribution of *Testudo graeca* adapted from Giménez et al. [33] and location of the study area.

Morocco is a demographically young country (45% of people are under 25 years) and has long been the home to foreign residents, who represented 0.25% of the total Moroccan population in 2014 (40% of these were Europeans, mainly French; [31]). The urbanization process developed in the country has caused some rural depopulation. Generally, education level in rural areas is lower than in urban ones, especially among females; only 28% of women received education in primary school in the period 1991–1998, while this percentage increased to 47% in the case of men [32]. Poverty ranged between 12% and 27% in the cities and rural areas, respectively, in the period 1984 to 1999; and the urban unemployment rate is the 22%. Our survey was conducted in the region of Rabat, which is home to 1.4 million habitants. In particular, we focused on the city of Rabat (578,519 habitants in 2014; Direction Regionale de Rabat-Sale-Kenitra) and the smaller village of Sidi Allal El Bahraoui (14,846 habitants in 2014; Direction Regionale de Rabat-Sale-Kenitra), which are separated by 38 km (see Figure 1).

The city of Rabat concentrates political and administrative services and it is inhabited by over 12,000 foreigners (15% of the Moroccan foreign community; [34]). Population structure by gender is similar in Rabat and Sidi Allal El Bahraoui and there is a predominance of the 15–59 age group, but this group is over-represented in comparison with the whole nation [32]. The percentage of people employed is slightly higher than in the rest of the country (19% unemployment rate in Rabat). The economic activity of Rabat is mainly related to the service sector (administrative function 39.4%, services 17.0%, and commerce 14.7%) and its inhabitants’ housing ranges from villas and modern houses in the new areas to modest houses in the peripheral districts [34].

The principal economic activity in Sidi Allal El Bahraoui is agricultural production and its inhabitants live mainly in districts dominated by modest flats and houses, although there are also some modern houses in the peripheral areas. Sidi Allal El Bahraoui is located inside the Maamora forest and Rabat is found at approximately 14 km from this cork oak (*Quercus suber*) forest (Figure 1). The Maamora forest has been in decline since 1955 owing to human pressure [35], and it is considered to be one of the areas with the highest density of Mediterranean spur-thighed tortoise documented to date throughout the species’ distribution range [21]. In Morocco, wild animals, including tortoises, are traded in pet shops, but more frequently in open markets [36]. In both cities and villages, market shops are often grouped together by theme. Wild animals are generally sold in herbalists’ shops, which also offer domestic animals, herbs, spices, and medicinal products. Each market usually has a few stalls selling animals caught in the wild, with the number varying according to the city [36]. The Mediterranean spur-thighed tortoise is by far the most frequently traded species on Moroccan markets; 94% of the 2113 wild animals surveyed by Bergin and Nijman [36] on 48 market stalls were *Testudo graeca* and their price depends on their size, with a small individual costing around 1US$.

### 2.2. Questionnaire Surveys

We carried out a survey based on a questionnaire, which was distributed by hand to residents throughout the day (e.g., 10 am to 17 pm) between Monday and Friday (i.e., working days) in the five main streets of Rabat and Sidi Allal El Bahraoui during the autumn of 2018. These precise locations were considered as representative of the study areas, as most of the residents concentrate around them on a weekday basis. We approached all the adult members (i.e., over 18 years) of the general public who walked through these streets and asked them to participate in a survey concerning tortoises as pets. Therefore, our target group comprised adults over 18 years of age who lived in the rural and urban areas surveyed (Supplementary Material, Table S1). The response rate was 98.9% (total n = 485). The participants responded to the questionnaires in person in either Arabic (in the case of those who had lived in Morocco all their lives: local participants) or in English (in the case of foreigners who were living in Morocco when the survey was conducted). All the foreigners were associated with the urban group owing to their low representativeness in smaller Moroccan villages (Supplementary Material, Table S2). A native Arabic speaker translated the questionnaires to Arabic and helped us during the interviews with local participants in order to avoid misinterpretations of the content of the questionnaire. All the respondents were assured that the contents of the questionnaires would be anonymous and confidential. The answers were analyzed all together and in agreement with Law nº 9-08 of 18th February 2009 concerning personal data protection in Morocco.

The standardized questionnaire (Supplementary Material, Figure S1) included both binary (yes/no) and Likert scale (from 1, total disagreement, to 5, total agreement) questions. A total of 21 questions were arranged in four main themes. The first group of questions dealt with general perceptions about tortoises (e.g., whether they are pets that can be obtained from a shop or from the forest, or whether they are wild species inhabiting the forest and have an ecological value), and also with the frequency with which Moroccan residents keep tortoises as pets. Here, we also evaluated the respondents’ preferences regarding pets by showing them pictures of a dog, a cat, a rabbit, a Mediterranean spur-thighed tortoise, a fresh water turtle, and a fish. We assessed the respondents’ preferences in terms of the tortoises’ age classes. Differences may be expected because (1) “babies” may be associated with cuteness, (2) adults may be viewed as stronger owing to the higher mortality of juveniles, and (3) adults are able to breed. We asked non-owners (i.e., those participants who had never owned a tortoise) if they would have any preferences regarding the tortoises’ age (juvenile or adult) and how they would potentially obtain a tortoise in the future (buying it from a shop or market stall or taking it from the Maamora forest). The objective of the second group of questions was to assess the participants’ biological and ecological knowledge of the species. In particular, we asked them about the longevity, sex differentiation, reproduction and feeding habits of tortoises, the role of Maamora forest as a home to tortoises, or the status of the species regarding its conservation. The third block of the questionnaire addressed tortoise owners’ personal experience with tortoises as pets (non-owners were not questioned about this). This part included questions about tortoises kept at home as pets (hereafter, captive tortoises). For example, we asked owners about the age of captive tortoises, estimated according to their carapace length (CL) (juvenile if <100 mm CL and adults if >100 mm CL; [37]) and their origin (e.g., captured in the wild or bought from either the market stall or a shop). We also inquired whether they had exchanged tortoises with other people, which is a common practice, especially among foreigners who spend some years in Morocco, whether their tortoises had bred in captivity and the number of times that this had occurred (1, 2–4, >4), and whether they had released the tortoise and if so, where (e.g., released into the forest or transferred to other people). We also asked about where the owner of the captive tortoise lived (i.e., a flat or a house with a garden), how long the captive tortoise had been living with them (e.g., less than a year and over three years), and tortoise care (e.g., a yearly checkups by a vet and obtaining information on tortoises by means of, for example, books or the Internet). Here, we also asked about the number of captive tortoises owned per person. We considered three categories: 1, 2–4, and >5 tortoises. This allowed us to estimate adult and juvenile tortoise ranges and the number of tortoises owned per person. For calculations, we assigned two tortoises to the minimum and four to the maximum in the category 2–4, while six tortoises were assigned to the >5 category. The percentage of tortoises was calculated according to the frequency of each category. In addition, we estimated roughly the number of captive tortoises in the study area. To do this, we first calculated the number of households in Rabat and Sidi Allal El Bahraoui, using the total human population living in these localities (Direction Regionale de Rabat-Sale-Kenitra) and the household size average in Morocco, which was 4.6 in 2014 [38]. Second, we estimated the number of captive tortoises per household in Rabat and the neighboring village, using the percentage of urban and rural people who kept captive tortoises and the number of tortoises owned per person according to the results of our survey. We did not include foreign people for this calculation because we did not know the household size for this group of people and because most foreigners remain in Morocco for less than 4 years, which makes any extrapolation difficult.

Finally, we recorded the respondents’ demographic information, such as age, gender, children, home, nationality, how long they had resided in Morocco, education level, urban/rural, type of home (i.e., house/flat), whether their work was related to nature, whether they spent their free time in natural surroundings, and whether they had any other pet at home (a full copy of the questionnaire is provided in the Supplementary Material).

### 2.3. Statistical Analysis

Generalized linear models (GLM) with a binomial distribution and a “logit” link function were used to explore the sociodemographic differences (i.e., nationality, age, gender, children home, urban/rural, type of home, education level, whether their work was related to nature, whether they spent their free time in natural surroundings, and whether they owned any other pet) between those who did and did not own tortoises. The most parsimonious model was selected using a backward stepwise procedure based on the Akaike information criterion (AIC; [39]). The Chi-square tests were used to assess differences between rural, urban local, and foreign residents in terms of the origin of their tortoises (from the wild, trade, other people, or found in the street), the captive breeding of tortoises, the tortoises’ destination (wild or street release, given to other people, and lost or dead), home in which the tortoise was kept (i.e., flat or house), how long the tortoise had been kept in captivity, whether checkups were conducted by vets, and whether they attempted to obtained any information about captive tortoises. We also used Chi-square tests to assess differences between owners and non-owners’ preferences regarding tortoises’ ages and origins. All the statistical analyses were performed using R 3.6.1 software [40].

## 3. Results

A total of 480 people participated in our survey, of whom 55% were tortoise owners (either because they had a tortoise at the moment of the interview or because they had had a tortoise in the past). The percentage of participants who owned other pets was considerably lower: 14% owned dogs, 9% cats, and less than 2% other pets (rabbits, turtle and fish). Our statistical model reported that the profile of a tortoise owner corresponded to a mature Moroccan person (over 30 years of age) living in a house with a garden in either urban or rural areas who often also owned other pets (Table 1).

The owners’ and non-owners’ perceptions and knowledge of tortoises were generally similar (Table 2). In contrast, some of the differences between rural and urban people and between locals and foreigners were pronounced. For example, rural people’s ecological and biological knowledge of the species was higher than that of urban people (Table 2).

### 3.1. Pet Preference and General Perceptions of Tortoises

Between half and three quarters of the people who answered the questionnaire preferred dogs to other pet species, including tortoises (Table 2). People had lower preferences for rabbits and freshwater turtles than for tortoises in all the groups, the only exception being that foreigners had a higher preference for turtles than for tortoises (Table 2). Some people liked adults because of their strength, but the majority preferred juveniles owing to their cuteness. The percentage of rural people who preferred cute juveniles and breeding adult tortoises was double that of local and foreign people living in Rabat (Table 2).

When we asked non-owners about their preference in the case of obtaining a tortoise, most of them responded that they would take it from the forest, while less than a quarter would buy it from shops or market stalls (Table 3). This pattern was particularly strong in the village, where three quarters of the participants responded that they would get the tortoise from the forest; in contrast, this percentage was significantly lower in the case of foreigners (rural people vs. foreigners: X^2^ = 6.7, *p* < 0.05, n = 121; Table 3), who would buy one from a shop much more frequently (rural people vs. foreigners: X^2^ = 11.9, *p* < 0.05, n = 121; Table 3). In terms of tortoise age preference, a little more than one third of the non-owners preferred juveniles, while half of them did not have any preference (Table 3).

More than half of the people interviewed perceived the tortoise as a pet. Notwithstanding, most people also viewed the tortoise as a wild species, which was especially evident among rural people, although some foreign and urban people even attributed it an ecological value (Table 2). In addition, over one-third of the participants in our study believed that it is common for people in Morocco to keep tortoises as pets. Nevertheless, most foreigners who had tortoises did not share this perception (Table 2). Most people considered houses with gardens as adequate homes for tortoises, while only less than half of the participants viewed flats as adequate for tortoises. The perceived inadequacy of flats as homes for tortoises was particularly high among foreigners (Table 2).

### 3.2. Biological and Ecological Knowledge of Tortoises

Nearly three quarters of the rural people considered that the Maamora forest was the home of tortoises, while this percentage fell to two quarters in the case of the two groups of urban people (Table 2). Near half of the rural people knew that the tortoise is a threatened species, while only a third of those living in the city and a quarter of the foreigners were aware of this.

Between half and three quarters of the participants in this study had appropriate knowledge of the longevity and feeding habits of this species. Knowledge regarding the sexual dimorphism and reproduction habits of the species was generally lower; nevertheless, it was higher among rural than urban people (Table 2).

### 3.3. Captive Tortoises

Most owners had obtained their tortoises from wild populations (42%, n = 264) followed by from other people, house heritage, market stalls, or shops; very few owners had found their tortoises roaming freely in the street (Table 4). The percentage of rural people who had obtained their tortoises from wild populations was double that of local people living in Rabat, which was as much as almost 10 times higher than the percentage of foreigners who had captured their tortoises in the forest (rural people vs. urban locals: X^2^ = 10.8, *p* < 0.05, n = 220; rural people vs. foreigners: X^2^ = 22.1, *p* < 0.05, n = 147; Table 4). An opposite pattern was found for the percentage of people who had received their tortoise/s from other people or had them in the garden of the rented house (tortoise exchange, rural people vs. urban locals: X^2^ = 11.1, *p* < 0.05, n = 220; rural people vs. foreigners: X^2^ = 9.6, *p* < 0.05, n = 147; house heritage, rural people vs. foreigners: X^2^ = 15.9, *p* < 0.05, n = 147; Table 4).

Nearly a quarter of the owners had tortoises that bred in captivity; differences between tortoises owned by rural and urban people were not observed in this respect. The number of times that tortoises bred in captivity was higher when they were owned by local people than by foreigners (Table 4).

Nearly half of the owners reintroduced their tortoise into the wild and almost one quarter stated that the tortoise had died. The remaining options were chosen less frequently (Table 4). The percentage of rural people who reintroduced their tortoise/s into the wild was double that of urban people (rural people vs. urban locals: X^2^ = 10.2, *p* < 0.05, n = 205; rural people vs. foreigners: X^2^ = 9.9, *p* < 0.05, n = 130). In contrast, the proportion of urban locals who gave their tortoise/s to other people was significantly higher than that of local people living in the village (rural people vs. urban locals: X^2^ = 8.8, *p* < 0.05, n = 205).

A little more than half of the owners had the tortoises in houses with a garden; this was significantly more frequent for foreigners than for rural people (rural people vs. foreigners: X^2^ = 4.7, *p* < 0.05, n = 71; Table 4). In addition, most owners had had their tortoises for less than a year (Table 4). The pertinence of the tortoise was significantly higher for foreigners than for rural people (rural people vs. urban foreigners: X^2^ = 6.8, *p* < 0.05, n = 147; Table 4).

In general, very few people took the tortoise to the vet for an annual checkup (Table 4). Half of the owners obtained information about their tortoises by reading books or searching on the Internet (Table 4).

The total number of tortoises owned by our participants ranged between 568 and 828 (an average of 2.1–3.1 tortoises/person; see methods for details of the calculations); nearly half of these tortoises were owned by rural people (Table 4). Most owners had only one tortoise (46%) or between two and four tortoises (48%). Most captive tortoises were juveniles (57%, n = 264). Local people (both urban and rural) owned a higher proportion of juveniles (60%) than foreigners (45%). According to our estimation, the number of tortoises kept in captivity in Rabat would be over 83,000 individuals and in the neighboring village over 3000 tortoises (Table 4).

## 4. Discussion

Understanding the implications of the trading and collection of wildlife regarding animal welfare and species conservation is important because, as the use of wildlife as pets grows, the associated impacts are likely to increase [41]. In the particular case of reptiles, the trading and collection of pets raises several ethical concerns regarding animal welfare and also poses a major risk for the conservation of some species, whose decline may lead to environmental degradation [8,42]. Our study may constitute a good example of the potential impacts of pet collection on animal welfare and conservation, as an intensive harvest of Mediterranean spur-thighed tortoises is evidenced (see also [43,44]).

### 4.1. Species Conservation

The use of live reptiles destined for pet markets is considered one of the major threats to the conservation of rare and endemic species. In this respect, 355 species of reptiles were intentionally targeted by collectors, including 194 non-CITES-listed species [45]. International trade in certain groups or species has been documented, e.g., 96 species of chameleons in Africa [46] or 155 species of turtles in China [47]. Conservation strategies have focused mostly on the control of trade in the species by means of regulations, but policy enforcement in several countries, such as Thailand, Indonesia, India, and Morocco, has been demonstrated to be inefficient regarding preventing the overexploitation of species [5,45]. The case of Mediterranean spur-thighed tortoises in Morocco is particularly illustrative, since our study demonstrates that owing tortoises as pets is very common in Rabat and its surroundings, although the species is legally protected. Importantly, most captive tortoises, particularly juveniles, are collected for non-commercial purposes in the forest, which probably puts wild populations in jeopardy. In Morocco, it is very common for local people to spend Sundays in the forest having picnics, i.e., cooking the traditional “tajine”. In the Maamora forest, where most tortoises are captured, a weekly presence of 30,000 visitors has been reported on sunny days [35]. This means that such visitors may very often encounter a tortoise in the forest, and the likelihood of tortoise collection is, therefore, high. Tortoise abundance is, in fact, higher in the protected areas of the Maamora forest than in the non-protected areas, where populations are unbalanced [21], and this may, at least partially, be a consequence of the tortoise owners’ higher preference for juveniles. In this line, Tiar et al. [22] reported the collection of tortoises from wild populations with non-commercial objectives as a common practice that might also have demographic consequences for Mediterranean spur-thighed tortoise populations in Algeria.

The release of captive tortoises into the wild, which would, according to our results, appear to be a frequent activity in Morocco, may also have detrimental consequences for native populations, including the transmission of pathogens and the alteration of tortoises’ demographic structure, sex ratio, and population size [48]. In other words, releasing pet tortoises into the forest would not only not solve the problem caused by tortoise collection, but would also aggravate it.

According to our study, the percentage of tortoises that die in captivity in Rabat and its surroundings is similar to the number reported in the UK by Lawrence [49], although the causes of mortality in our study area might be different to those observed in the UK (i.e., difficulties during hibernation). The mortality of captive tortoises might play a role in the species’ demography: adult mortality is higher in captivity than in the wild (10% in wild populations; [50]), and this might force owners to collect more tortoises from the forest once their pet tortoises die. In addition, our findings show that there is also a significant flow of tortoises from one person to another, and foreigners are especially involved in transferences of this nature, as they frequently spend only a few years in the country. The number of tortoises owned per person reported by our survey was similar to that observed by Perez et al. [20] in southern Spain and higher than in north Algeria (1.4 tortoise per person; [22]). This, together with our estimate of over 80,000 animals in Rabat and with previous findings that revealed that the Mediterranean spur-thighed tortoise is the most frequently sold species on Moroccan market stalls [36], suggest that the risk of overharvesting the tortoise population in the Maamora forest might, therefore, be considerable.

In conclusion, tortoise collection for non-commercial uses might affect demographic parameters of wild populations (e.g., fecundity), likely reducing juvenile recruitment and hence population growth rate. It can also contribute to disease spread [51] and possibly to outbreeding depression if the released animals contact among genetically distinct populations. As a result, tortoise collection might cause local extinction in wild populations close to villages and large cities like Rabat [16]. Therefore, international conservation strategies might put more efforts on the reduction of tortoise collection, considering this threat alone as a major cause of the species population decline, likely as important as the pet trade [13] or habitat loss [35].

### 4.2. Animal Preference, Welfare, and Pet Knowledge

Our results show that people in our study areas prefer dogs and cats over tortoises, although the latter are more frequently owned than the former. Although domestic species are considered aesthetically pleasant, the reported preference for tortoises might be influenced by people’s previous attitude towards wildlife and their previous experience with and knowledge of the species [12]. In addition, this might be explained by the ease of obtaining a tortoise in our study areas and the very low cost of its maintenance both in terms of caring and feeding.

Owners’ knowledge of pet care has been shown to affect the human–animal relationship and the welfare of companion animals [52]. In the case of other domestic pets, a higher risk of pet relinquishment was associated with the owners’ limited knowledge of, for example, the ideal housing, diet, and medical care of their pets [53,54]. Animal welfare might be related to an animal’s nutrition, environment, health, behavior, and mental state [41,55], and owners should, therefore, ideally attain knowledge regarding all these issues. In the case of reptiles, the relationship between pet welfare and the owners’ knowledge has often been overlooked, probably because welfare impacts on pet reptiles are usually underreported [41]; assessing the welfare of reptiles is more difficult than for other animal groups, such as mammals, because reptiles do not show visible indicators of stress or disease [56]. In our study, tortoise owners rarely make use of available sources of information (e.g., books, the Internet, or a vet’s assessment) to familiarize themselves with the species’ habits and requirements, which may explain their limited knowledge of the species. This scarcity of owners’ knowledge regarding a basic understanding of their pets and their welfare has also been observed in studies developed in the USA and Canada with other pet reptile species [42,57,58,59]. In our study, most people did not know about tortoises’ reproduction habits, and even acknowledged difficulties in identifying the gender of the tortoise kept at home. This might compromise female tortoises’ survivorship if they are not provided with an adequate space in which to lay their eggs in the spring [37]. In addition, rural people did not consider that a house with garden or even an apartment is a poor environment for tortoises, although tortoise’s movements are likely to be restricted in such places in comparison to their natural range, as occurs with lizards and snakes (e.g., [42]). The exception was people’s relatively high knowledge of tortoise feeding habits, which might allow them adequate nutrition (including a high variety diet), thus contributing to the longevity of the species in captivity.

According to our results, the conditions of tortoises kept as pets were far from being ideal as described in the Moroccan markets by Bergim and Nijman [36]. Tortoises in captivity are likely affected by discomfort in thermoregulation, if they do not have access to sun or to appropriate floor as occurs in the flats and some houses where tortoises are kept in captivity. Injuries are usually reported in the case of tortoises that share homes with dogs or from garden machines as a result of tortoise hiding behavior. Diseases might be expected in tortoises kept as pets as it has been reported elsewhere in this tortoise species; a mutualistic relationship such as nematode–tortoise, often turns to a parasitic relationship harming the tortoise body condition [59]. The isolation or the continuous presence of a possible predator like dogs might cause fear, anxiety, and distress on the tortoises, regarding the rarity of living in a flat—very different from their natural environment—[60] and the continuous feeling of being susceptible of being preyed.

In addition, in our study the length of tortoise ownership is generally less than three years. This is very short when considering the long lifespan of tortoise species, and much shorter than for other pets like dogs or cats in Europe [61], but is in line with that of other wild pets, high percentages of which end up being abandoned or in animal care centers owing to their overly demanding requirements [62]. Moreover, there is almost a complete absence of tortoise veterinary care in Rabat and its surroundings, which contrasts with the frequent care of other domestic pets like cats, dogs or rabbits in, for example, North America [63].

### 4.3. Differences between Rural and Urban People

Rural people had a higher perception of tortoises as a wild species, demonstrated a higher biological and ecological knowledge of the species, and were less attached to captive tortoises (i.e., most owned the tortoises for less than a year). These differences may be related to the fact that rural people come into far more contact with the tortoise habitat during their daily work activities, such as farming or the harvesting of camomile, truffles, acorns, and cork [35], and during their leisure activities (e.g., playing games with their children). Moreover, both rural and urban people acquire tortoises from wild populations, as was found in the case of other Testudinidae species, which are sold for food rather than as pets [30]. Nevertheless, rural people obtain tortoises from and release them into the forest more frequently, although they seem to be more conscious than urban people that the Mediterranean spur-thighed tortoise is a threatened species. The relationship between the owners’ knowledge of tortoises and their concern about potential tortoise overexploitation was not within the scope of this study. However, in the future it would be interesting to explore the possibility that increasing owners’ knowledge could improve their attitudes towards tortoise conservation, as this has not occurred in other contexts (e.g., [64]).

### 4.4. Recommendations

Despite the fact that people usually prefer domestic pets like cats and dogs, as our study confirms, the truth is that wild species are also often kept as pets; our results showed that many people in Morocco own a Mediterranean spur-thighed tortoise. Motivations for acquiring wild pets and the diverse roles of actors that move beyond the pet trade and collection could be studied in much greater depth [25,65]. The non-economic motivations that drive different actors to participate in both wildlife collection and trade could, in turn, particularly inform more appropriate, fair, and effective conservation actions [66]. Increasing people’s knowledge of the overexploitation caused by the pet trade and collection through educational campaigns is often requested [52,67]. New ways of reaching pet tortoise owners are required in Morocco, owing to their low potential regarding access to information sources, such as books and the Internet, and their limited contact with veterinarians. In this respect, a recent study revealed that consumers’ demand for wild pet species may be reduced by up to 40% if pet owners are provided with information about either legal issues or the risk of zoonotic diseases associated with buying/keeping certain species [68]. In addition, it is essential that education campaigns of this nature target the appropriate people [26,27,28]. Our study reveals that, in our context, awareness efforts should be aimed at mature Moroccan people (over 30 years of age) who live in a house with a garden and also own other domestic pets, given that they are the people who mostly keep tortoises as pets in the study area. In fact, these people might have been in contact with tortoises for longer and might be influenced by a longer tradition of keeping tortoises as pets [23], and owners of domestic pets are probably more open to the idea of keeping wild species as pets [25,69]. In addition, special efforts should be made to raise awareness among rural people that collecting tortoises from the forest is illegal and detrimental to wild populations. Finally, but of no less significance, the population monitoring of species threatened by the pet trade and collection and the provisioning of scientific knowledge to overcome uncertainties to prevent overexploitation is crucial [47]. In conclusion, conservation strategies might consider the role played by non-commercial collection as one of the possible major causes of the Mediterranean spur-thighed tortoise decline.

## Figures and Tables

**Table 1:** Results of generalized linear model (GLM) explaining sociodemographic tortoise owner profile. Only the most parsimonious models (i.e., those with the lowest Akaike information criterion (AIC)) are shown. Significant differences between people ranging from 31 to 50 and over 50 years are observed (Z value: 2.109, *p* <0.05). See Table S3 (Supplementary Material) for the full list of models tested.

**Model Predictor**	**Estimate**	**Std Error**	**Z Value**	***p* Value**	**AIC**
Nationality: local	0.6696	0.2583	2.592	<0.05	639.4
Age: 31–50	0.228	0.211	1.08	0.28	
Age: over 50	0.8418	0.3217	2.617	<0.05	
Home: house	0.8284	0.2213	3.742	<0.05	
Pet owner	0.4400	0.1948	2.259	<0.05	
**Model Predictor**	**Estimate**	**Std Error**	**Z Value**	***p* Value**	**AIC**
Nationality: local	0.7474	0.2647	2.824	<0.05	639.4
Age: 31–50	0.2488	0.2121	1.173	0.24	
Age: over 50	0.8921	0.3247	2.748	<0.05	
Home: house	0.8479	0.2226	3.809	<0.05	
Urban/rural	−0.2877	0.2013	−1.43	0.15	
Pet owner	0.4701	0.1965	2.392	<0.05	
**Model Predictor**	**Estimate**	**Std Error**	**Z Value**	***p* Value**	**AIC**
Nationality: local	0.7428	0.2647	2.806	<0.05	639.7
Age: 31–50	0.3873	0.2377	1.63	0.1	
Age: over 50	1.1036	0.3632	3.039	<0.05	
Children	−0.3007	0.2282	−1.317	0.19	
Home: house	0.8898	0.2257	3.943	<0.05	
Urban/rural	−0.2824	0.2018	−1.4	0.16	
Pet owner	0.4780	0.1969	2.427	<0.05	

**Table 2:** Tortoise owners’ and non-owners’ pet preferences, perceptions, and knowledge. The percentages are highlighted with different intensities of colors according to their level: dark grey = over 67%, medium grey = 34%–66% and light grey = less than 33%.

	Rural People				Urban Locals				Foreigners				Total	
	Tortoise Owner		Non-Owner		Tortoise Owner		Non-Owner		Tortoise Owner		Non-Owner			
Pet preference (%)	n = 103		n = 87		n = 117		n = 95		n = 33		n = 34		n = 469	
Dog	74.8		64.4		45.3		47.4		66.7		73.5		59	
Cat	51.5		44.8		54.7		46.3		30.3		35.3		47	
Tortoise	26.2		13.8		23.9		9.5		15.2		5.9		18	
Rabbit	22.3		28.7		17.9		13.7		12.1		5.9		19	
Turtle	7.8		5.7		8.5		5.3		30.3		11.8		9	
Fish	7.8		13.8		24.8		17.9		30.3		38.2		19	
Tortoise preference (%)	Disagree	Agree	Disagree	Agree	Disagree	Agree	Disagree	Agree	Disagree	Agree	Disagree	Agree	Disagree	Agree
Cute baby	18.4	68.9	19.5	51.7	25.6	47.9	24.2	46.3	33.3	33.3	32.4	29.4	24.0	51.0
Breeding adult	36.9	38.8	36.8	26.4	26.5	20.5	45.3	12.6	45.5	6.1	50.0	5.9	37.0	22.0
Strong adult	33.0	32.0	26.4	32.2	26.5	28.2	44.2	16.8	24.2	30.3	44.1	11.8	33.0	26.0
Pet perception (%)														
As a pet: present	29.1	63.1	36.8	49.4	23.9	58.1	18.9	53.7	24.2	42.4	41.2	47.1	28.0	55.0
As wild species	6.8	88.3	3.4	87.4	11.1	69.2	8.4	76.8	18.2	60.6	17.6	58.8	9.0	77.0
Morocco: tortoise as a pet	9.7	78.6	14.9	70.1	7.7	81.2	6.3	80.0	3.0	48.5	8.8	73.5	9.0	75.0
As a pet: field removal	24.3	69.9	32.2	50.6	10.3	76.1	21.1	69.5	30.3	36.4	29.4	41.2	22.0	63.0
As wild: ecological value	11.7	72.8	6.9	69.0	6.8	70.9	7.4	73.7	6.1	81.8	5.9	79.4	8.0	73.0
Pet knowledge (%)														
Maamora forest: home	14.6	72.8	12.6	72.4	17.9	47.9	14.7	53.7	9.1	51.5	8.8	44.1	10.0	59.0
Threatened species	38.8	40.8	32.2	46.0	22.2	35.0	25.3	40.0	24.2	24.2	11.8	32.4	28.0	38.0
Flat adequacy	41.7	46.6	51.7	31.0	38.5	41.0	44.2	34.7	75.8	15.2	64.7	8.8	47.0	35.0
House adequacy	9.7	83.5	20.7	70.1	12.0	72.6	12.6	78.9	15.2	72.7	14.7	64.7	14.0	75.0
Longevity	15.5	61.2	12.6	58.6	11.1	58.1	4.2	54.7	3.0	57.6	2.9	73.5	14.0	59.0
Age at reproduction	14.6	40.8	5.7	26.4	12.0	15.4	7.4	6.3	3.0	12.1	5.9	5.9	9.0	20.0
Feeding: vegetables	10.7	79.6	14.9	64.4	11.1	77.8	3.2	82.1	6.1	81.8	8.8	82.4	10.0	77.0
Feeding: herbs	3.9	86.4	1.1	89.7	4.3	78.6	3.2	82.1	6.1	66.7	2.9	73.5	3.0	82.0
Sexual dimorphism	11.7	60.2	8.0	51.7	9.4	32.5	8.4	27.4	6.1	24.2	14.7	17.6	10.0	40.0
Reproduction habits	12.6	66.0	5.7	63.2	5.1	47.9	5.3	46.3	3.0	30.3	2.9	35.3	7.0	52.0
Aestivation	22.3	49.5	11.5	48.3	19.7	27.4	13.7	22.1	27.3	21.2	8.8	17.6	17.0	34.0

**Table 3:** Tortoise age and origin preference of those people who did not have a tortoise. Differences between rural people and urban locals (P1) and rural people and foreigners (P2) according to Chi-square tests; *p*-values are shown: ns (not significant), * (*p* < 0.05).

	Total (n = 216)	Rural People (n = 87)	Urban Locals (n = 95)	Foreigners (n = 34)	P1	P2
Tortoise age						
Juvenile tortoise (%)	38	37	39	41	ns	ns
Adult tortoise (%)	12	13	8	18	ns	ns
No preference (%)	50	50	53	41	ns	ns
Tortoise origin						
Wild population (%)	57	74	52	26	ns	*
Trade: shop (%)	25	13	26	53	ns	*
Trade: market stall (%)	18	13	22	21	ns	ns

**Table 4:** Results of the surveys in the study area, in urban and rural areas for local and foreign people. Minimum and maximum numbers of captive tortoises were calculated according to the ranges of the questionnaire (tortoises kept: between two and four were assigned to two as a minimum and four as a maximum, while over four was assigned to five). The same procedure was followed to estimate breeding times. Differences between rural people and urban local (P1) and rural people and foreigners (P2); ns (statistically not significant) and * (*p* < 0.05). Flat and house were assigned only to people who currently had tortoises, and not those who had had them in the past.

	n	Total	Rural People	Urban Locals	Foreigners	P1	P2
People who owned a tortoise (%)			43	37	20	ns	ns
Captive tortoises		568–828					
Juvenile tortoise		323–467	146–204	126–190	51–73		
Adult tortoise		245–361	99–137	85–127	61–97		
Tortoises per person		2.1–3.1	2.4–3.3	1.8–2.7	2.5–3.9		
Number of households			3227	125–765			
Estimate of number of tortoises kept as pets			3330–4579	83,759–125,639			
Origin	264						
Wild population (%)		42	70	32	4	*	*
Trade: shop (%)		5	4	6	5	ns	ns
Trade: market stall (%)		10	7	15	5	ns	ns
Exchange with other people (%)		20	8	28	31	*	*
House heritage (%)		17	9	12	45	ns	*
Recover from street (%)		6	2	7	10	ns	ns
Captive breeding (%)	264	20	23	17	20	ns	ns
Breeding times		90–120	32–48	30–44	21–25		
Destination	234						
Release: forest (%)		44	67	29	13	*	*
Release: street (%)		3	2	4	3	ns	ns
Transfer (%)		14	5	21	20	*	ns
Lost (%)		17	11	20	30	ns	ns
Died (%)		22	15	26	34	ns	ns
Home and Care	264						
Flat * (%)	94	46	65	39	13	ns	*
House * (%)	94	54	35	61	87	ns	*
Pertinence time: 1 year (%)		50	58	48	34	ns	ns
Pertinence time: 2–3 years (%)		30	29	32	30	ns	ns
Pertinence time: over 3 years (%)		20	13	20	36	ns	*
Vet care (%)		3	1	3	7	ns	ns
Documented (%)		51	47	56	43	ns	ns

## Data Availability

The datasets generated during and/or analyzed during the current study are available from the corresponding author on reasonable request.

## Supplementary Materials

The following are available online at https://www.mdpi.com/2076-2615/10/2/265/s1, Table S1: Questionnaire completed by participants, Table S2: Demographic characteristics, Table S3: List of models tested.
