# Supplementary material for: Implications for Conservation of Collection of Mediterranean Spur-Thighed Tortoise as Pets in Morocco: Residents’ Perceptions, Habits, and Knowledge
Source: Animals (Basel). 2020 Feb 7;10(2):265. doi: 10.3390/ani10020265 (PMC7070921; doi:10.3390/ani10020265)
Supplement: Supplementary file 1 [file animals-10-00265-s001.pdf]

animals

Supplementary Material

Table S1. Questionnaire sheet filled in by the people surveyed

Dear Sir/Madam:

My name is Amalia Segura González, and I am a researcher at Castilla-La Mancha University (UCLM), Spain. I am currently researching humans' attitudes towards Mediterranean spur-thighed tortoises when kept as pets. This research is part of my PHD, whose objective is to view the situation of this tortoise in Rabat and the surrounding areas from different perspectives.

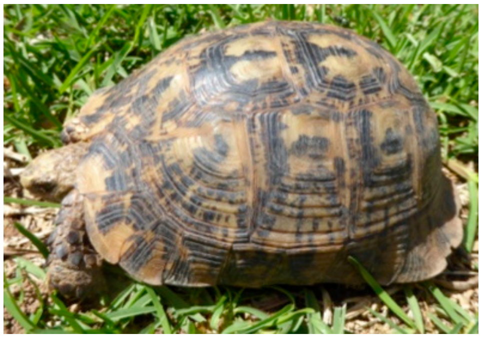

I would be very grateful if you could spend 10 minutes filling in the attached questionnaire.

The responses are anonymous and will be analyzed all together. The information contained in the questionnaires will comply with Law nº 9-08 of 18th February 2009 regarding Personal data protection in Morocco.

Your help is essential for this research.

If you have any questions, do not hesitate to contact me at: testudo.graeca.morocco@gmail.com

Thank you for your valuable collaboration.

Amalia Segura González
Researcher
Castilla-La Mancha University
Ciudad Real, Spain

Questionnaire

Please mark with an x, as appropriate:

1. PET PERCEPTION AND PREFERENCE

1.1. Please select the level to which you agree with the following statements, taking into account that 1 means in total disagreement and 5 means in total agreement

	Totally disagree	Disagree	Neither agree nor disagree	Agree	Totally agree
The tortoise is a pet species that people buy in shops as presents	1	2	3	4	5
The tortoise is a wild species that lives in the forest	1	2	3	4	5
In Morocco, people keep tortoises as pets	1	2	3	4	5
The tortoise is a pet species that people take from the country to their homes	1	2	3	4	5
The tortoise is a wild species with an ecological value	1	2	3	4	5

1.2. Which of these animals would you like to own as a pet? (First option)

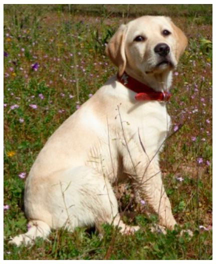	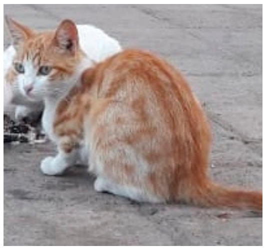	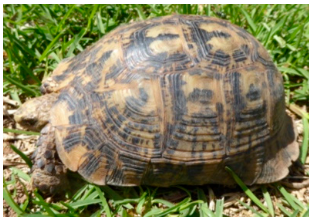
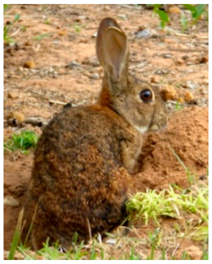	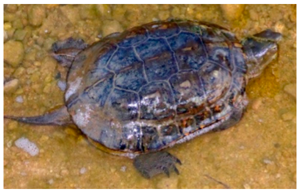	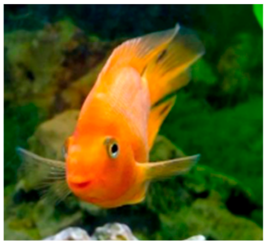

Which of these animals would you like to own as a pet? (Second option)

1.3. Do you have any preference as regards adult or baby tortoises? Please, select your level of agreement with the following statements

	Totally disagree	Disagree	Neither agree nor disagree	Agree	Totally agree
I prefer babies because they are cute	1	2	3	4	5
I prefer adults because they can have babies	1	2	3	4	5
I prefer adults because they are bigger and stronger	1	2	3	4	5

1.4. Please select your level of agreement with the following statements

	Totally disagree	Disagree	Neither agree nor disagree	Agree	Totally agree
Tortoise develop adequately in a flat	1	2	3	4	5
Tortoise develop adequately in a house with a garden	1	2	3	4	5
A tortoise's home is Maamora forest	1	2	3	4	5
The tortoise is a long-living animal that can reach 40 years of age	1	2	3	4	5
The tortoise is a threatened species in Morocco	1	2	3	4	5

1.5. Please select your level of agreement with the following statements

	Totally disagree	Disagree	Neither agree nor disagree	Agree	Totally agree
Tortoises reproduce when they are over 6 years old	1	2	3	4	5
Tortoises feed on fruit and vegetables	1	2	3	4	5
Tortoises feed on a diversity of herbs	1	2	3	4	5
The female tortoise is bigger than the male	1	2	3	4	5

Female tortoises lay their eggs in the soil in spring	1	2	3	4	5
Tortoises spend the summer buried in soil	1	2	3	4	5

2. PERSONAL EXPERIENCE OF TORTOISES

2.1. Do you own a pet tortoise?

Yes ☐ ☐ Not now, but I did in the past ☐ No

If yes, how many tortoises do you have at home (please select one of the following options):

<100 mm of carapace length: 1 ☐ 2-4 ☐ >4 ☐

>100 mm of carapace length: 1 ☐ 2-4 ☐ >4 ☐

2.2. If you were thinking of getting a tortoise, would you?

☐ Buy it from a shop ☐ Buy it from the souk ☐ Get it from the forest

2.3. If you were thinking of getting a tortoise, would you?

☐ Get an adult ☐ Get a baby ☐ I don't have a preference

If you don't have one at present, or you haven't in the past jump to question 3

2.4. How did you get the tortoise? Select one of the options (or several if you have more than one)

☐ It was already at home

☐ I found it in the street

☐ I took it from the forest

☐ I bought it from a shop

☐ I bought it from the souk

☐ Someone gave it to me

2.5. Has the tortoise bred at home?

Yes ☐ ☐ No

If yes, how many times: 1 ☐ 2-4 ☐ >4 ☐

2.6. If you have had a tortoise in the past but not now, what happened to it?

☐ I gave it to other people

☐ I released it in the forest

☐ I released it in the street

☐ It died

☐ It escaped

2.7. For how long have you owned a tortoise/s?

<1 year ☐ 1-3 years ☐ >3 years ☐

2.8. What resources do you use to obtain tortoise care information?

Pet store staff ☐ Books ☐ Internet ☐ Veterinaries ☐
None ☐

2.9. How often do you take your tortoise for regular veterinary checkups?

Never ☐ Once per year ☐ More than once per year ☐

3. PERSONAL PROFILE

3.1. What is your nationality?

3.2. What is your gender?

☐ Male ☐ Female

3.3. What is your age range?

☐ 18-30 ☐ 31-50 ☐ >50

3.4. Do you have children?

☐ Yes ☐ No

3.5. What is your level of education?

☐ No studies

☐ Elementary studies

- University studies

3.6. How long have you been living in Morocco?

- <1 year
- 1-4 years
- >4 years
- All my life

3.7. Where do you live?

- I live in a flat in the village
- I live in a house with a garden in the village
- I live in a flat in the city
- I live in a house with a garden in the city

3.8. Is your job related to nature?

- Yes
- No

3.9. Do you spend your free time in contact with nature?

- Yes
- No

3.10. Are you in contact with the village?

- I have family or close friends living there
- I work in the village
- Although I don't have any contacts in the village, I go often there
- I never go to the village

3.11 Do you own any pets at home?

Dog Cat Fish Rabbit Turtle

Other: which one?

Table S2. Demographic characteristics (%). *The foreigners were predominantly European (80%), with a few Americans (10%) and South Africans (10%).

	Tortoise owner		Non-owner	
	Urban	Rural	Urban	Rural
Gender, age & children				
Female (%)	53.4	30.1	59.7	37.9
Male (%)	46.6	69.9	40.3	62.1
With children (%)	47.2	54.4	47.3	49.4
Age: 18-30 (%)	38.5	28.2	42.6	41.4
Age: 31-50 (%)	49.1	38.8	48.8	49.4

Age >50 (%)	12.4	22.3	8.5	9.2
Education level				
University (%)	70.8	16.5	64.3	31.0
Elementary (%)	26.7	69.9	34.9	58.6
No studies (%)	2.5	13.6	0.8	10.3
Home				
House (%)	44.1	34.0	17.8	33.3
Flat (%)	55.9	66.0	82.2	66.7
Relation with nature				
Work (%)	9.9	54.4	10.9	46.0
Like (%)	87.0	97.1	89.9	94.3
Pets				
Other pets (%)	42.2	72.8	46.5	55.2
Nationality				
Local (%)	72.7	96.1	73.6	80.5
Foreign* (%)	27.3	4.9	26.4	19.5

Table S3. List of models tested.

**Model formulation**	**AIC**
Nationality+Gender+Age+Children+Education+Home+Urban+Work+Nature+Pet holder	648.1
Nationality+Gender+Age+Children+Home+Urban+Work+Nature+Pet holder	644.9
Nationality+Gender+Age+Children+Home+Urban+Nature+Pet holder	642.9
Nationality+Gender+Age+Children+Home+Urban+Pet holder	641.5
Nationality+Age+Children+Home+Urban+Pet holder	639.7
Nationality+Age+Home+Urban+Pet holder	639.4
Nationality+Age+Home+Pet holder	639.4

© 2019 by the authors. Submitted for possible open access publication under the terms and conditions of the Creative Commons Attribution (CC BY) license (http://creativecommons.org/licenses/by/4.0/).
